# Plasma extrachromosomal circular DNA is a pathophysiological hallmark of short‐term intensive insulin therapy for type 2 diabetes

**DOI:** 10.1002/ctm2.1437

**Published:** 2023-10-20

**Authors:** Zhe Xu, Junyu He, Peng Han, Peiji Dai, Wei Lv, Nian Liu, Liyi Liu, Liehua Liu, Xiaoguang Pan, Xi Xiang, Hanbo Li, Fangfang Ge, Shan Gao, Zhihong Liao, Yonglun Luo, Yanbing Li

**Affiliations:** ^1^ College of Life Sciences University of Chinese Academy of Science Beijing China; ^2^ Department of Endocrinology The First Affiliated Hospital, Sun Yat‐Sen University Guangzhou China; ^3^ Lars Bolund Institute of Regenerative Medicine Qingdao‐Europe Advanced Institute for Life Sciences BGI Research Qingdao China; ^4^ Department of Biochemistry and Molecular Biology School of Basic Medicine Qingdao University Qingdao China; ^5^ Scientific Research Center The Seventh Affiliated Hospital of Sun Yat‐sen University Shenzhen People's Republic of China; ^6^ BGI Research Shenzhen China

**Keywords:** diabetes, extrachromosomal circular DNA, short‐term intensive insulin therapy

## Abstract

**Background:**

Extrachromosomal circular DNA (eccDNA) has emerged as a promising biomarker for disease diagnosis and prognosis prediction. However, its role in type 2 diabetes remains unexplored.

**Objective:**

To investigate the characteristics and dynamics of circulating eccDNAs in newly diagnosed type 2 diabetes mellitus (T2DM) patients undergoing short‐term intensive insulin therapy (SIIT), a highly effective treatment for inducing long‐term glycemic remission.

**Methods:**

We conducted Circle‐Seq analysis on plasma samples from 35 T2DM patients at three time points: pre‐SIIT, post‐SIIT, and 1‐year post‐SIIT. Our analysis encompassed the characterization of eccDNA features, including GC content, eccDNA length distribution, genomic distribution, and the genes in eccDNAs.

**Results:**

Following SIIT, we observed an increase in plasma eccDNA load, suggesting metabolic alterations during therapy. Notably, a correlation was identified between eccDNA profiles and glycemia in T2DM, both quantitatively and genetically. Our analysis also revealed the frequent presence of metabolism‐related genes within T2DM plasma eccDNAs, some of which spanned gene exons and/or fractions.

**Conclusion:**

This study represents the first report of cell‐free eccDNA in T2DM and underscores a compelling association between cell‐free eccDNA and profound glycemic changes. These findings highlight the potential of eccDNAs as crucial players in the context of T2DM and glycemic control.

## Graphical abstract

This study presented the first plasma extrachromosomal circular DNA (eccDNA) landscape of type 2 diabetes mellitus treated with short‐term intensive insulin therapy. Metabolism‐related genes are unevenly distributed in eccDNAs from low and high glycemia patients. The cell‐free eccDNA is significantly correlated with changes in glycemia and long‐term glycemic control.

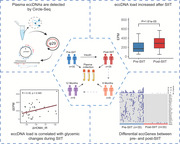

## 1 INTRODUCTION

Type 2 diabetes mellitus (T2DM) affects approximately 537 million adults globally and leads to high mortality due to severe complications.^1^ Continuous T2DM treatment and research improvements are needed and would significantly improve public health. Short‐term intensive insulin therapy (SIIT) stands as a forefront approach in the quest to reverse T2DM.^2^ It improves islet β‐cell functions and insulin sensitivity by reducing glucotoxicity and lipo‐toxicity, hence achieving a sustained normalization of blood glucose levels over the long term.^3^

The long‐term diabetic reversal effect of SIIT exhibits significant patient heterogeneity, and its underlying mechanism largely remains unknown. T2DM is closely related to insulin resistance and islet malfunction.^4^ It has been demonstrated that the implementation of SIIT for a duration of 1 to 2 weeks, utilizing continuous subcutaneous insulin infusion (CSII) induces the recovery and maintenance of β‐cell functions.^5^ Intensive insulin should be administered with caution in frail patients who are prone to hypoglycemia and contraindicated in those with obvious acute or chronic complications, for example, diabetic retinopathy, heart failure, or kidney disease. SIIT usually was carried out during hospitalization.^6^ However, due to the inaccessibility of the pancreatic tissues, the identification of plasma biomarkers to monitor the treatment and pancreatic β‐cell functions becomes imperative. Additionally, other physiological changes that accompany the therapeutic outcomes are required to be recognized.

Circulating nucleic acids (RNA and DNA) have been explored as potential biomarkers for monitoring the status of T2DM.^7^ Successful examples of cell‐free DNA (cfDNA) include circulating unmethylated cfDNA of insulin coding genes, insulin DNA fragments, and circulating miRNA genes.^8^, ^9^, ^10^, ^11^, ^12^, ^13^ Extrachromosomal circular DNAs (eccDNAs) are circular DNA molecules originating from chromosomes. It is frequently detected in body fluids, such as plasma and urine.^14^, ^15^, ^16^ The unique double‐stranded circular structure of eccDNA makes it more resistant to exonucleases in cells than linear DNA.^15^ In addition to cellular eccDNAs, cell‐free eccDNA has been found in urine, serum, and plasma, with a size relatively larger than linear cfDNA.^14^, ^15^, ^17^ We thus hypothesize that plasma eccDNA may reflect the status of cells affected by hyperglycemia and related metabolic disorders, suggesting a promising new biomarker for T2DM.

In the present study, we analyzed the plasma eccDNA in 35 newly diagnosed T2DM (New‐DM) patients before (pre‐SIIT) and after SIIT (post‐SIIT) treatment, as well as 1 year after the cessation of anti‐hyperglycemic medication (1 year‐SIIT). This study marks the first comprehensive report on the eccDNA landscape in SIIT for T2DM, further highlighting the relationship between eccDNA and hyperglycemia.

## 2 METHODS

### 2.1 Ethical review, patient recruitment and sample processing

The present study was approved by the academic research department of the First Affiliated Hospital of Sun Yat‐sen University (NO.174‐1^18^) and the Institutional Review Board (IRB) of BGI‐Shenzhen (BGI‐IRB 22007). Before initiating study procedures, all enrolled patients provided informed consent. The eligible New‐DM participants had a duration of T2DM of less than 1 year and had never been on any hypoglycemic drugs. T2DM was diagnosed according to the criteria of the World Health Organization (1999). Additional inclusion criteria included an age range of 18−70 years old, a body mass index between 20 and 35 kg/m^2^, and glycosylated haemoglobin (HbA1c) ≥ 7.0%. The patients with acute diabetic complications, obvious microvascular and macrovascular complications, systemic infection, malignant tumours, or pregnancy were excluded. Thirty‐five patients participated in the study and received SIIT using CSII therapy. The primary clinical characteristics of the participants are displayed in Table 1.

**TABLE 1:** Clinical data of newly diagnosed type 2 diabetes patients (*n* = 35) before and after short‐term intensive insulin therapy (SIIT).

	Pre‐SIIT (*n* = 35)	Post‐SIIT (*n* = 35)	*p*‐Value
Age (years)	52.4 ± 7.1		
Sex (male/female)		24/11	
FPG (mmol/L)	11.05 ± 2.93	5.77 ± 1.01	<0.001
HbA1c (%)	10.56 ± 1.94	9.02 ± 1.77	<0.001
HOMA‐β	18.13 ± 17.34	51.81 ± 62.45	<0.001
HOMA‐IR	3.41 ± 1.8	1.53 ± 0.98	<0.001
AUC_glu_	33.41 ± 6.77	22.7 ± 3.98	<0.001
AUC_ins_	33.24 ± 32.92	58.02 ± 39.98	<0.001
AUC_ins/glu_	0.97 ± 1.39	2.91 ± 1.92	<0.001

*Note*: Continuous parametric data are presented as means ± SD. Continuous nonparametric variables are presented as medians (interquartile ranges). And categorical data were presented as proportion.

Abbreviations: AUC_glu_, the area under the glucose curve during a standard food‐load test; AUC_ins_, the area under the insulin curve during a standard food‐load test; AUC_ins/glu_, AUC_ins_/AUC_glu_ ratio; FPG, fasting plasma glucose; HbA1c, glycated haemoglobin; HOMA‐β, homeostasis model assessment of insulin secretion; HOMA‐IR, homeostasis model assessment of insulin resistance; SIIT, short‐term intensive insulin therapy; *p* < 0.05 was considered significant.

### 2.2 The intervention of the patients

Blood samples were collected for baseline pre‐SIIT evaluation. CSII was administered to achieve normal glycemic levels, with fasting glycemic levels at 4.4–6.0 mmol/L and 2‐hour postprandial glucose levels at 4.4–8.0 mmol/L. The initial total insulin dose ranged from 0.4 to 0.8 IU/kg/day. Half of this dose was provided as basal insulin evenly over 24 h, and the other half was assigned equally before three meals. Capillary blood glucose (CBG) level was tested eight times daily (before and 2 h after three meals, before bedtime, and at 3 AM midnight). The Insulin dosage was adjusted according to the CBG level. The CSII treatment was continued for another 1 week after the normal glycemic levels were achieved. Subsequently, blood samples for post‐SIIT evaluation were collected on the day following the discontinuation of CSII.

After SIIT, the 35 patients were visited every 3 months for follow‐up information. Nine patients who underwent lifestyle intervention without hypoglycemic drug administration, and were included in further investigation. At the final visit at 1 year after therapy, glycemic remission was defined as FPG <7 mmol/L and HbA1c<7%. Non‐remission was defined as FPG ≥7 mmol/L or HbA1c≥7% at the 1‐year visit.

### 2.3 Isolation of plasma DNA

A 5 mL peripheral blood sample was collected for eccDNA analysis. Plasma was obtained through centrifugation within three hours after blood sampling. Three hundred microliter of plasma samples together with 20 uL proteinase K were incubated at 55°C for 15 min. The total plasma DNA, consisting of both linear and eccDNA, was isolated from the reaction mix from the previous step using the MGI Easy Circulating DNA Extraction Kit (MGI‐BGI). DNA concentration was measured with a Qubit Fluorometer (Invitrogen).

### 2.4 eccDNA purification and amplification

To enrich eccDNA, we removed the plasma linear DNA by incubating the sample with 20 units of Plasmid‐Safe DNase (Epicenter) over 12 h at 37°C in a 50 uL reaction system. Then we purified the digestion products with 90 uL Ampure XP beads and elute the DNA in 28 uL nuclease‐free water.

Then we used the purified eccDNA as template for rolling circle amplification (RCA) with highly processive phi29 polymerase. The RCA reaction was carried out and incubated at 30°C for 24 h.

### 2.5 Library preparation and sequencing

1 ug of phi29‐amplified DNA products was fragmented using Covaris LE220 to generate 300–500 bp DNA fragments. Then 80 ng of the fragmented DNA was subjected to library preparation using the MGIEasy DNA Library Preparation Kit (MGI‐BGI). We evaluated the content and quality of the sequencing library with Bioanalyzer 2100 (Agilent). We sequenced the library by paired‐end 150 bp using an MGISeq‐2000 sequencing machine.

### 2.6 Preprocessing of raw sequencing data

The raw sequencing reads were quality‐checked using fastQC (v0.11.3) (https://www.bioinformatics.babraham.ac.uk/projects/fastqc). Then the quality information of all samples was collected by MultiQC (v1.10.1).^19^ Those read sequences produced in FASTQ format were pre‐processed through fastp (v0.21.0).^20^ The remaining reads that passed all the filtering steps were counted as clean reads. An index of the reference genome (GRch38.p13) was built using BWA (v0.7.12),^21^ and clean reads were aligned to the reference genome using BWA‐MEM.

### 2.7 Detection of eccDNA

To detect eccDNAs from the aligned data, we applied Circle‐Map (v1.1.4) software (https://github.com/iprada/Circle‐Map) to detect the coordinates of each eccDNA.^22^ To improve the accuracy of eccDNA detection, several filtering steps were performed as previously described. The specific settings were as follows: (1) the number of split reads is greater than 2, (2) the score of the circle is more than 200, and (3) the mean base coverage within the ccDNA detection coordinates is greater than the standard deviation of the base coverage vector, (4) coverage increase at the start/end coordinate by more than 0.3, (5) the fraction of bases not covered by reads within the ccDNA detection coordinates is lower than 0.1.

### 2.8 Genomic and sequence features of eccDNAs

After mapping the overall population of plasma‐derived eccDNA in the human genome, the numbers of eccDNA molecules whose starting positions map to genomic sequences were obtained. The theoretical distribution of plasma eccDNA was predicted based on the percentage of the genome covered. The normalized genomic coverage of plasma eccDNA was then calculated using the following formula:

$$\begin{array}{ccc} & & eccDNA\;per\;million\;mapping\;reads\;\\ & & \;=\frac{eccDNA\;counts}{total\;mapping\;reads\;number}\;*1e06 \end{array}$$

We used the “observed/expected ratio of genomic elements” for statistical analysis of seven major classes of genomic elements (i.e., 3′UTR, 5′UTR, CpG island, exon, Gene2KbD, Gene2KbU, and intron), and the “observed/expected ratio of genomic elements” was calculated according to the following formula:

$$\begin{array}{ccc} & & \mathrm{observer}/\mathrm{expected}\;\mathrm{ratio}\;\mathrm{of}\;\mathrm{genomic}\;\mathrm{elements}\;\\ & & =\frac{\mathrm{Percentage}\;\mathrm{of}\;\mathrm{unique}\;\mathrm{eccDNA}\;\mathrm{falling}\;\mathrm{in}\;a\;\mathrm{certain}\;\mathrm{type}\;\mathrm{of}\;\mathrm{elements}}{\mathrm{Percentage}\;\mathrm{of}\;\mathrm{the}\;\mathrm{length}\;\mathrm{of}\;\mathrm{that}\;\mathrm{element}\;\mathrm{over}\;\mathrm{the}\;\mathrm{length}\;\mathrm{of}\;\mathrm{the}\;\mathrm{whole}\;\mathrm{genome}} \end{array}$$

Additionally, using bedtools (v2.25.0),^23^ we counted the number of reads that map to specific repeat elements. The normalized mapping ratio was calculated as the percentage of reads that mapped to a specific repeat element divided by the percentage of the specific repeat element present in the nuclear genome.

### 2.9 Genome annotation of eccDNA fragment junction sites

To calculate the amount of each gene to produce eccDNA, we defined the abundance of eccDNA on a gene using the number of eccDNA on each gene and the gene length. The eccDNA abundance was calculated as follows:

$$\begin{array}{ccc} & & \mathrm{eccDNA}\;\mathrm{abundance}\;\mathrm{for}\;\mathrm{gene}\;\\ & & \;=\frac{\mathrm{the}\;\mathrm{number}\;\mathrm{of}\;\mathrm{eccDNA}\;\mathrm{types}\;\mathrm{from}\;\mathrm{one}\;\mathrm{gene}\;}{\mathrm{gene}\;\mathrm{length}\;\times \;\sum_{n=1}^{i}\left(\frac{\mathrm{the}\;\mathrm{number}\;\mathrm{of}\;\mathrm{eccDNA}\;\mathrm{types}\;\mathrm{from}\;\mathrm{one}\;\mathrm{gene}}{\mathrm{gene}\;\mathrm{length}}\right)}\\ & & \;\times \;1.0e06 \end{array}$$

Genes with differential eccDNA abundance were then identified based on a Wilcoxon rank‐sum test, and differential genes with significant eccDNA abundance were filtered out using the threshold of *p* < 0.05 and |log2 FC| ≥ 1. Gene Ontology (GO) and Kyoto Encyclopedia of Genes and Genomes (KEGG) enrichment analyses were implemented by the WEB‐based gene set analysis toolkit (http://www.webgestalt.org/).

### 2.10 Validation of eccDNA

The eccDNA validation was performed by outward PCR and Sanger sequencing, and PCR primers were listed in Table S1. Each 30 uL PCR reaction system included 50 ng of phi29‐amplified DNA products, 500 nM primer, 15 uL NEBNext High‐Fidelity 2X PCR Master Mix (NEB), and PCR reaction for 40 cycles. All reactions were performed accompanied by a non‐template control. The PCR products were tested by agarose (2%) gel electrophoresis, and the target products were recovered by the QIAEX II Gel Extraction Kit, and sent for Sanger sequencing.

### 2.11 Calculate changes in blood glucose and beta cell function

To quantify changes in blood glucose and beta cell function following SIIT treatment, several parameters were calculated using specific formulas:

(1) The HOMA‐β was calculated by 20×fasting insulin levels (FINS)/(FPG‐3.5). (2) The HOMA‐IR was evaluated by FPG×FINS /22.5. (3) The insulin Secretion‐Sensitivity Index‐2 (ISSI‐2) was calculated by AUCins/glu multiplied by the Matsuda index via a standard food load test. The AUCins/glu was the ratio of the area under the curve for insulin (AUCins) and the area under the curve for glucose (AUCglu). The Matsuda index was evaluated by 10 000/square root of (Ins0 × Glu0) × (mean glucose × mean insulin during a standard food load test).

The venous blood samples were drawn for measurement of glucose and insulin at fasting and 10, 20, 30, 60, 90, and 120 min following ingestion of the standard food load. The standard food contained 7 g of protein, 10.8 g of fat, 42.7 g of carbohydrates and 1.38 g of sodium, providing a total energy content of 300 kcal.

To study the percentage changes in blood glucose and beta cell function after SIIT treatment, a series of delta values was defined to represent the percentage change after SIIT treatment. The formula is as follows:

$$\Delta Value\;=\frac{PostValue-PreValue}{PreValue}\;\times 100$$

Here, “PostValue” represents the value after SIIT treatment, and “PreValue” represents the value before SIIT treatment.

### 2.12 Statistical analysis

All statistical tests were implemented using R‐4.1.1. The difference between the two groups was compared using the Wilcoxon rank‐sum test, where *p* < 0.05 indicated statistical significance.

## 3 RESULTS

### 3.1 Patients and clinical characteristics

To explore the correlation of plasma eccDNA and hyperglycemia in T2DM patients, we recruited 35 newly diagnosed (new‐DM) patients (see methods). Plasma samples were taken at the time of diagnosis (pre‐SIIT), after SIIT (post‐SIIT) and one year after the completion of SIIT (1 year‐SIIT). As shown in Table 1, SIIT led to significant improvements in glycemic profiles: The fasting plasma glucose (FPG) dropped from 11.05 ± 2.93 to 5.77 ± 1.01 mmol/L, while HbA1c fell from 11.05 ± 2.93 to 9.02 ± 1.77 % (*p* < 0.05). Additionally, there was a marked elevation in both HOMA‐β levels and integrated islet β cell functions (ISSI‐2) post‐SIIT (*p* < 0.05). We followed the 35 patients for 1 year after the completion of SIIT and further analyzed nine patients who had not taken any other anti‐diabetic medications. By the end of the follow‐up, we observed that three of them achieved remission, while the remaining six did not. Notable changes were observed in their clinical parameters, including FPG, HbA1c and AUGins/glu, as detailed in Table S2.

### 3.2 Genome‐wide mapping of plasma eccDNA in T2DM patients

**FIGURE 1:**
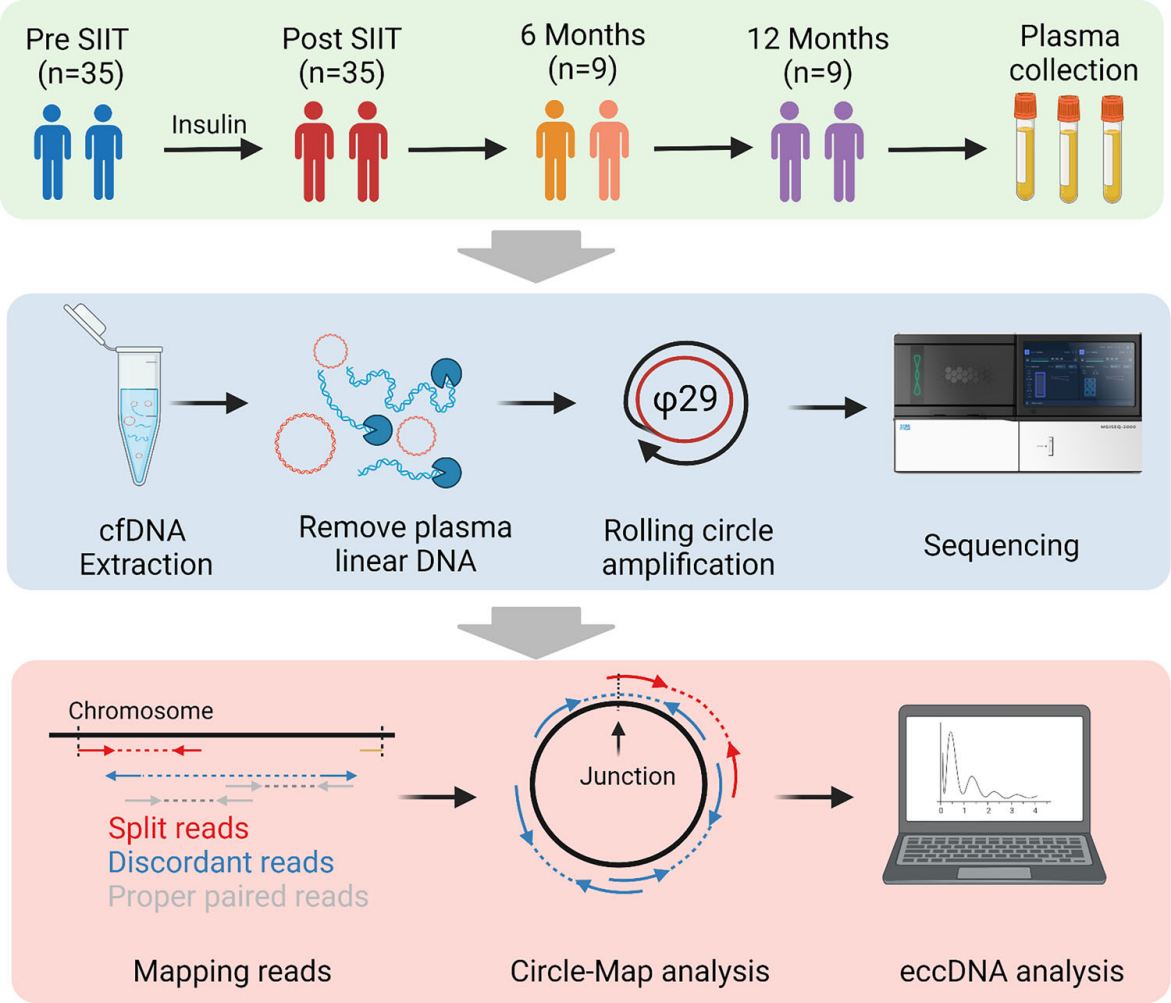
Experimental workflow of the present study and the modified Circle‐Seq method. Thirty‐five severe type 2 diabetes mellitus (T2DM) patients were recruited, and their plasma was collected at diagnosis and after short‐term intensive insulin therapy (SIIT). Total plasma cell‐free DNA, which contains both linear and circular DNAs, was isolated. Then linear DNA was removed by exonuclease, and the purified circular DNA was amplified by rolling circle amplification (RCA). The RCA products were sequenced by high‐throughput sequencing of pair‐end 150 bp. The Circle‐Map software was applied to identify extrachromosomal circular DNA (eccDNA) from sequencing data based on the recognition of split reads and discordant reads.

We then analyzed the plasma eccDNA in the pre‐SIIT and post‐SIIT samples by Circle‐seq.^22^ Our pair‐end high‐throughput sequencing yielded an average of 166 million reads per sample (Table S3). We applied Circle‐Map software to detect eccDNAs from sequencing data, identifying them based on discordant reads and split reads (Figure 1).^22^, ^24^ This led us to pinpoint 3,221,044 unique eccDNAs, with 1,815,505 from pre‐SIIT, and 1,405,539 from post‐SIIT samples.

**FIGURE 2:**
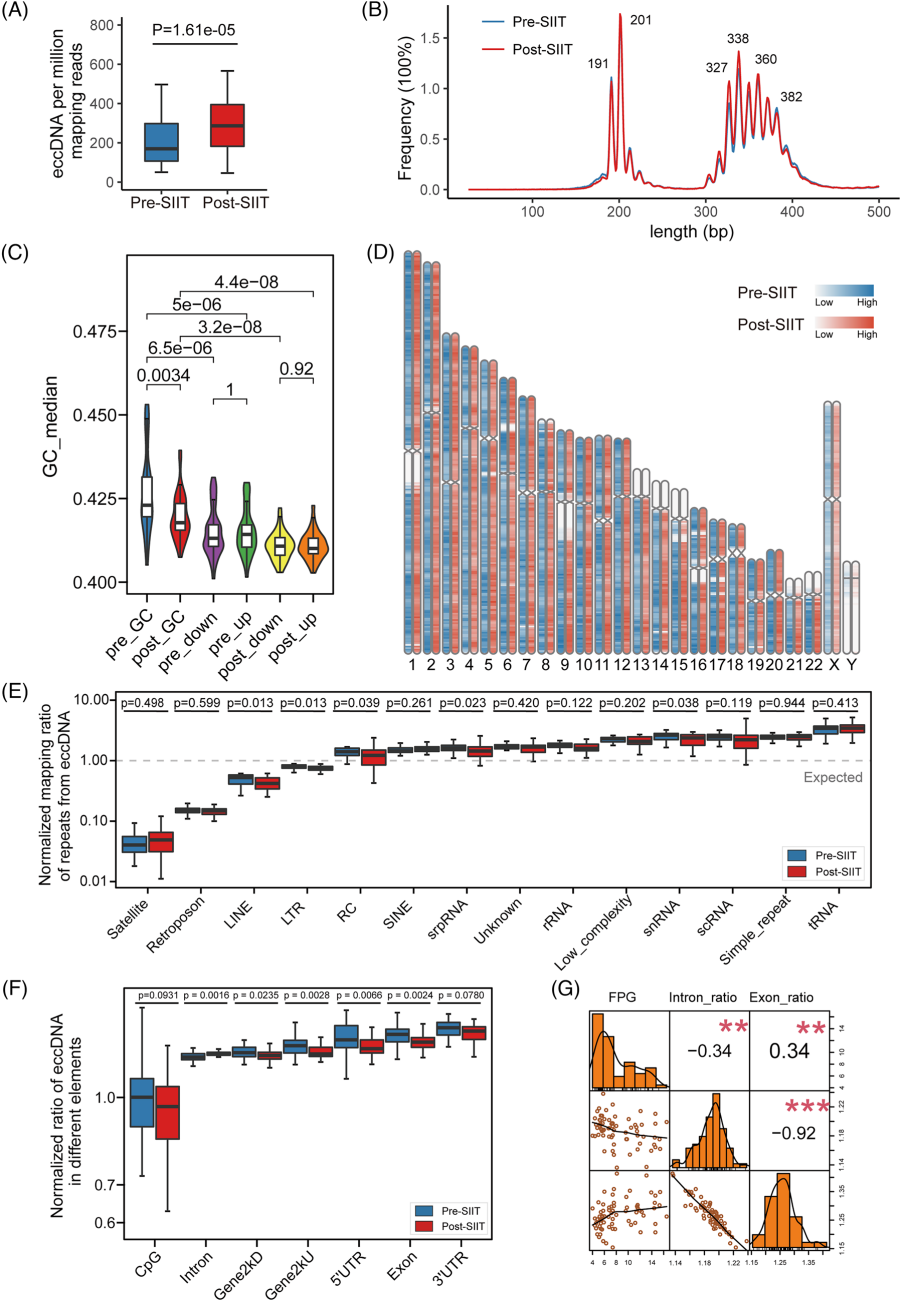
Comprehensive characterization of plasma extrachromosomal circular DNA (eccDNA) profiles in type 2 diabetes mellitus (T2DM). (A) Comparison of normalized eccDNA number between pre‐short‐term intensive insulin therapy (pre‐SIIT) (blue) and post‐SIIT (red) groups (Wilcoxon test). EPM, eccDNA count per million mapping reads (excluded reads mapped to mitochondrial DNA). (B) Length distribution of plasma eccDNA. Plasma eccDNA displays a multi‐modal distribution. (C) GC content distribution of plasma eccDNA and their downstream and upstream regions of equivalent length. (D) Genome‐scale distribution of plasma eccDNAs. The pre‐SIIT group and the post‐SIIT group are marked with blue and red respectively, and the shades of colour indicate the average number of eccDNAs per Mb on the chromosome. (E) Normalized mapping ratio of eccDNA reads aligned to repeats. SINE, short interspersed nuclear element; srpDNA, signal recognition particle DNA repeats; rDNA, ribosomal DNA repeats; tDNA, transport DNA repeats; scDNA, small conditional DNA repeats; snDNA, small nuclear DNA repeats; LTR, long terminal repeat; LINE, long interspersed nuclear element; RC, rolling circle repeats. (F) Normalized ratio of eccDNA in different genomic elements. UTR, untranslated region; Gene2kbD, 2 kb downstream of genes; Gene2kbU, 2 kb upstream of genes. (G) Correlation of fasting plasma glucose (FPG) with the normalized ratio of eccDNA in introns and the normalized ratio of eccDNA in exons.

To analyze the difference in the eccDNA load between pre‐SIIT and post‐SIIT samples, we normalize the eccDNA counts by million mappable reads (EPM), reducing the impact of sequencing depth on total eccDNA counts. Although the total unique eccDNA counts were lower in post‐SIIT, after normalization by EPM, the EPM in post‐SIIT was significantly higher than in pre‐SIIT (*p* = 1.61e‐05) (Figure 2A and Table S4).

Further analysis of eccDNA lengths indicated that most of the eccDNAs were shorter than 500 bp in both sample sets, aligning with prior studies (Figure 2B,C).^14^, ^15^ These eccDNAs mainly aggregated into two clusters of peaks of 201 and 338 bp (Figure 2B), which differed by an average of 10 bp in each cluster peak. Unlike the eccDNA length distribution in tissues, eccDNAs in plasma were not concentrated in one peak, but enriched in multiple peaks with consecutive intervals. The prominent peaks at 201 and 338 bp highly resembled the DNA structures of one and two nucleosomes. While most plasma eccDNAs were shorter than 500 bases, a small fraction exceeded 6000 bases in post‐SIIT (Figure S1C). Since eccDNAs were apoptotic products from cells, the increased EPM found in post‐SIIT suggests a mechanism of SIIT therapy by clearing out apoptotic cells in T2DM patients.^25^

### 3.3 Characteristics of plasma eccDNAs in T2DM

Having analyzed the quantity and size distribution of eccDNA, we proceeded to explore its intrinsic characteristics. The GC content of DNA is linked to its stability, and function, and is evolutionarily conserved.^26^ Previous studies on eccDNA in tissues, cell lines, and urine have shown that eccDNA is enriched in high GC content regions.^16^ In the plasma of T2DM patients, the peak of eccDNA GC content reached 42.3%, and the median GC content of the pre‐SIIT exceeded that of the post‐SIIT group (*p* = 0.0034) (Figure 2C and Table S4). Aligning with past observations, the GC content in the flanking sequences of these eccDNA fragments was markedly less than that of the fragments themselves (Figure 2C). The distinct GC content pattern of eccDNA implies an uneven distribution across chromosomes, underscoring the diverse origins of plasma eccDNA.

To provide deeper insights into the chromosomal origin of eccDNA, we aligned their sequences to chromosomes. We discerned a roughly uniform eccDNA distribution across each autosome, except for the low coverage of acrocentric chromosomes (13p, 14p, 15p, 21p, 22p, and Y). Additionally, regions surrounding the centromere of chromosomes 1, 9, and 16 displayed diminished eccDNA density (Figure 2D). Yet, the chromosomal distribution of eccDNA between pre‐SIIT and post‐SIIT remained strikingly consistent.

Repetitive elements compose 45% of the human genome. Notably, repetitive satellite elements and 5S rDNA are known to form eccDNAs in human cells.^27^ Our study revealed a pronounced derivation of eccDNA from long/short interspersed nuclear elements (LINE, *p* = 0.02; SINE, *p* = 0.039) in pre‐SIIT compared to post‐SIIT (Figure 2E and Table S5). An intriguing exploration of gene‐wide distributions of plasma eccDNAs displayed that the plasma eccDNA from post‐SIIT patients was significantly less enriched in exons (*p* = 0.0024), 5′UTR (*p* = 0.0066), Gene2kU (*p* = 0.0028), Gene2kD (*p* = 0.0235), as well as CpG (*p* = 0.0931) and 3′UTR (*p* = 0.078) compared to the plasma eccDNA from pre‐SIIT patients (Figure 2F). After SIIT treatment, the plasma eccDNA was more enriched in intronic regions (*p* = 0.0016).

To further address if the biased gene‐wide distribution of eccDNA in pre‐SIIT and post‐SIIT was correlated with patient status, we performed correlation analysis between intron and exon eccDNA proportion with FPG levels. Our analysis revealed a negative correlation between intron‐derived eccDNA proportions and FPG levels (Pearson's R = −0.34, *p* = 0.0041), whereas exon‐derived eccDNAs exhibited a positive correlation with FPG (Pearson's R = 0.34, *p* = 0.043) (Figure 2G). Collectively, our results showed that while the overarching chromosomal distribution of eccDNA remains consistent pre‐ and post‐SIIT, SIIT significantly affects the gene‐wide distribution of plasma eccDNA, representing a hallmark for T2DM and/or SIIT treatments.

### 3.4 Differential eccGenes between pre‐SIIT and post‐SIIT

To elucidate the dynamic changes of eccGenes (genes from linear chromosomes that form eccDNAs) during SIIT interference, we applied eccDNA abundance to evaluate the predisposition of a certain gene to form eccDNAs. Briefly, eccDNA abundance is defined as the number of eccDNAs derived from a gene and normalized by the gene length (see methods). We subsequently compared the relevance of the start and end junction sites, and identified a high correlation between these sites (R = 0.99, *p* < 2.2e‐16, Figure S1A), implying that similar outcomes can be obtained using either the start or end junction site.

**FIGURE 3:**
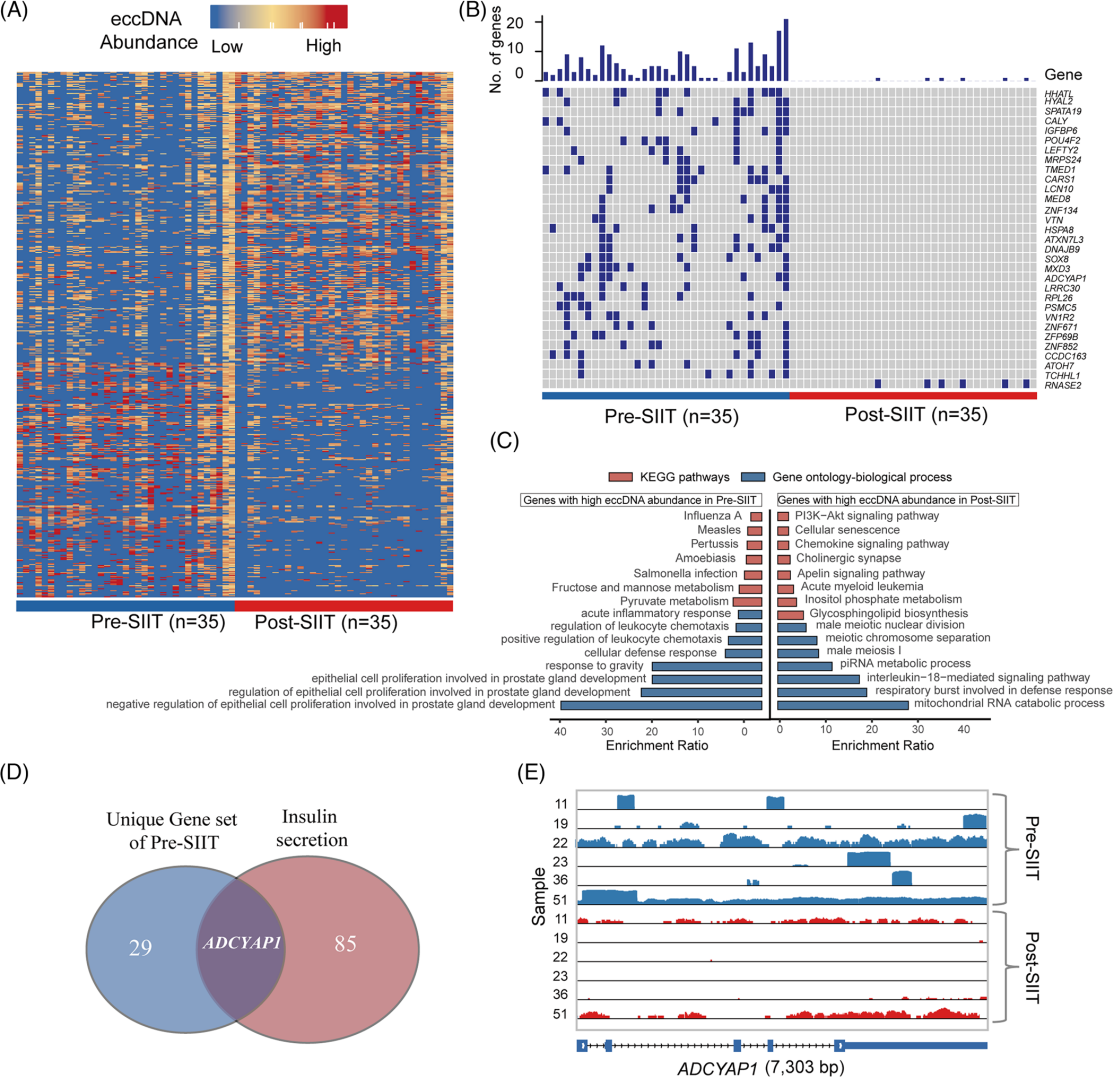
Differential plasma extrachromosomal circular DNA (eccDNA) patterns between pre‐short‐term intensive insulin therapy (pre‐SIIT) (blue) and post‐SIIT (red) groups. (A) Heatmap showing the differential eccGenes between pre‐ and post‐SIIT groups. The X‐axis and Y‐axis represent sample id and gene id respectively. 522 eccGenes presented differently between pre/post‐treatment groups by the Wilcox test. (B) The 31 unique eccGenes were chosen from 522 different eccGenes (Fisher's exact test). (C) Gene Ontology (GO) and Kyoto Encyclopedia of Genes and Genomes (KEGG) pathway analysis of differential eccGenes among pre/post‐SIIT group. (D) The intersection of 30 unique genes of the pre‐SIIT group and the Insulin secretion pathway. (E) IGV plots of eccDNA of ADCYAP1 gene between pre‐and post‐SIIT in six patients.

Differential analysis of eccDNA abundance showed that 522 genes were significantly different between pre‐SIIT and post‐SIIT (237 from pre‐SIIT, 285 genes from post‐SIIT) (Figure 3A and Table S6). The KEGG and GO enrichment of differentially presented eccGenes from the post‐SIIT group showed the inflammation and metabolism changes mainly responded to interleukin‐18‐mediated signalling pathway, histidine metabolism and inositol phosphate metabolism pathways. Meanwhile, in the pre‐SIIT group, they were enriched in the regulation of leukocyte migration, acute inflammatory response, regulation of leukocyte chemotaxis, pyruvate metabolism, and fructose and mannose metabolism pathways (Figure 3C).

To explore the direct relevance of plasma eccDNAs in T2DM, we employed more stringent criteria, focusing on genes that solely formed eccDNAs in either pre‐SIIT or post‐SIIT. By Fisher's exact test, we identified 30 eccGenes in pre‐SIIT whereas only 1 gene (*RNASE2*) in post‐SIIT (Figure 3B). Noteworthy, the *ADCYAP1* gene, a marker gene of islet β‐cell and involved in insulin secretion, derived eccDNAs in multiple samples.^28^ Through the IGV tool, we found that although there are reads enriched on *ADCYAP1* in the post‐SIIT sample, there is no junction site, so only derived eccDNA in six patients from the pre‐SIIT group and was absent in the post‐SIIT samples (Figure 3D, E and S3; Table S7). Meanwhile, genes involved in metabolism such as *IGFBP6*, *POU4F2*, *LEFTY2*, *CARS1*, *LCN10*, *HSPA8*, *DNAJB9*, *SOX8*, *ADCYAP1*, *TCHHL1* were also detected on eccDNAs. This underscores the propensity of metabolism‐centric genes to increasingly form eccDNAs during SIIT, potentially shedding light on the therapeutic efficacy of eccDNAs.

### 3.5 Exon‐carrying plasma eccDNAs are frequently derived from metabolism genes

It was reported that transcription‐active regions tend to generate more eccDNAs.^17^ Meanwhile, apoptosis and necrosis cells release most of the circulating DNAs.^25^, ^29^ As mentioned above, approximately 15% of the plasma eccDNAs were derived from the exons (Figure 2F), prompting the potential relevance to genomic functions.

**FIGURE 4:**
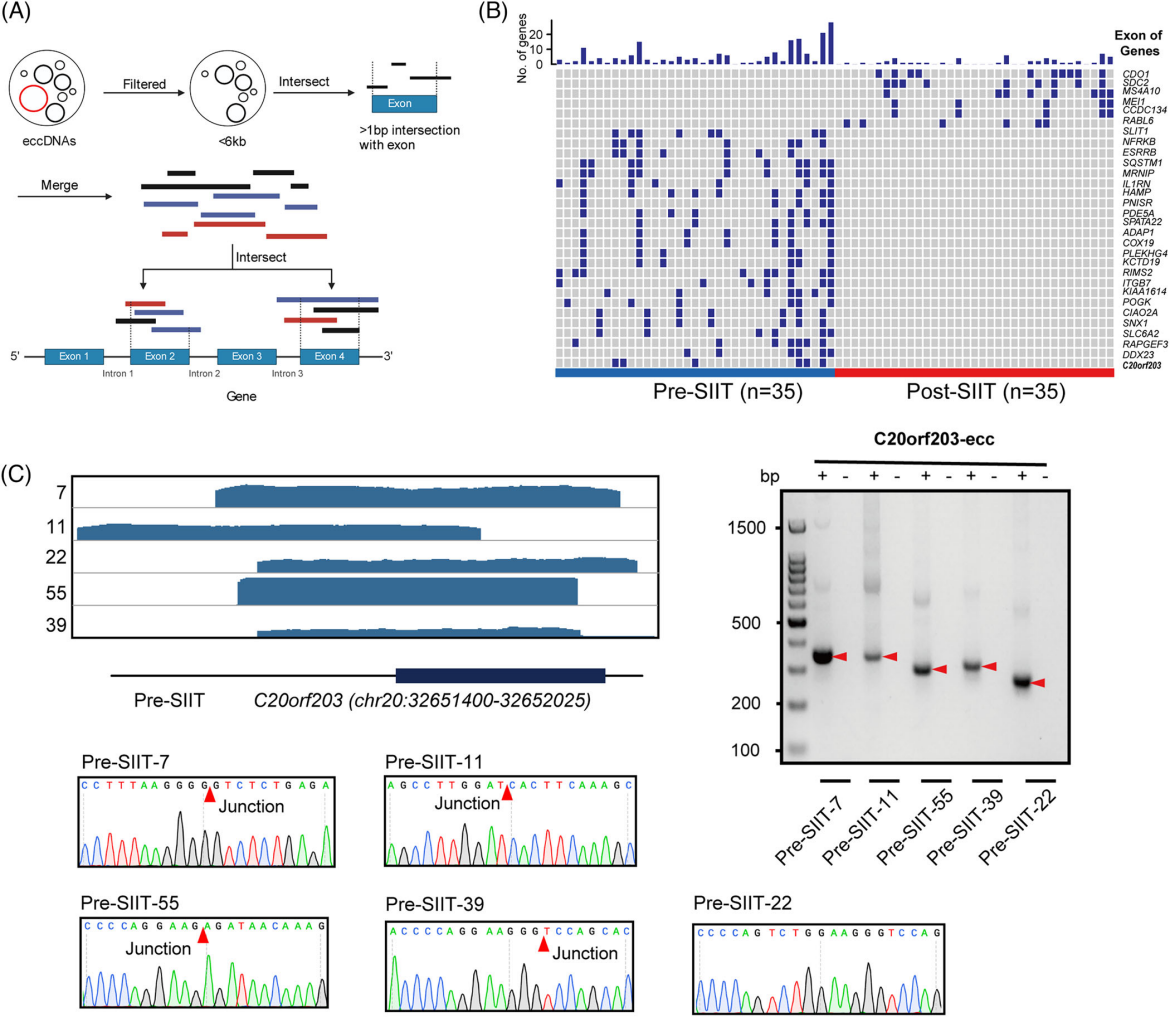
Validation of extrachromosomal circular DNAs (eccDNAs) in type 2 diabetes mellitus (T2DM) plasma samples. (A) The pipeline for eccDNA clusters analysis spans exon regions. (B) Top 30 unique exons presented in 135 unique eccGenes (Fisher's exact test). (C) Integrative Genomics Viewer (IGV) depicted for eccDNAs derived from the *C20orf203* gene. Each sample was marked as “Post/Pre‐short‐term intensive insulin therapy (SIIT)”, the black bar on the bottom line represents for exon region, the thick line means the exon region, and the thin line means the intronic region; PCR validation of eccDNAs from *C20orf203*, which frequently derived eccDNAs in multiple samples. Junction sites were obtained after the sequencing of PCR products.

To further elucidate the exon origin of eccDNAs, we merged eccDNAs from pre/post‐SIIT groups and created a cluster of eccDNAs which intersect with exons from the same gene. From these clusters, eccDNA sets spanning exons were selected for subsequent analysis. We identified unique sets of eccDNA spanning exons in both the pre‐SIIT and post‐SIIT groups (Figure 4A). In total, 522 exons were identified from eccDNAs in the pre/post‐SIIT groups (422 exons in the pre‐SIIT group and 100 exons in the post‐SIIT group) (Table S8). Among the 30 most frequently presented eccDNAs of this 522 exon‐carrying eccDNAs, we identified eccDNAs covering coding regions of well‐known metabolism‐related genes, including *SQSTM1*, *HAMP*, *PDE5A*, *PLEKHG4* and *ITGB7* (Figure 4B). Conclusively, here we underscore the notable connection between eccDNAs originating from exonic regions and metabolism‐related genes, particularly in the context of SIIT treatment.

To validate the acquired eccDNAs, we selected one eccDNA that coordinates within the exon sequence (ENSE00001543364.2, from the C20orf203 gene), and this circle was verified by outward‐PCR and Sanger sequencing (Figure 4C). Intriguingly, we also identified long eccDNAs spanning entire gene exons in our plasma eccDNA dataset. To verify the authenticity of these long eccDNAs, we selected 12 exon‐spanning eccDNAs (KRTAP22‐1, EPHA1‐AS1, H4C12, C10orf120, S100A6, OR1J2, H4C12, LCE3D, IFNA1, OR5L2, H2BC10 and OR4B1), with length ranging from 1 to 6 kb, and verified by outward‐PCR and Sanger sequencing (Figure S2). In conclusion, this exploration underscores a significant nexus between eccDNAs sourced from exonic zones and genes pivotal to metabolism, especially when observed in the milieu of SIIT treatment.

### 3.6 Potential role of eccDNAs as indicators of hyperglycemia and β‐cell function in T2DM

**FIGURE 5:**
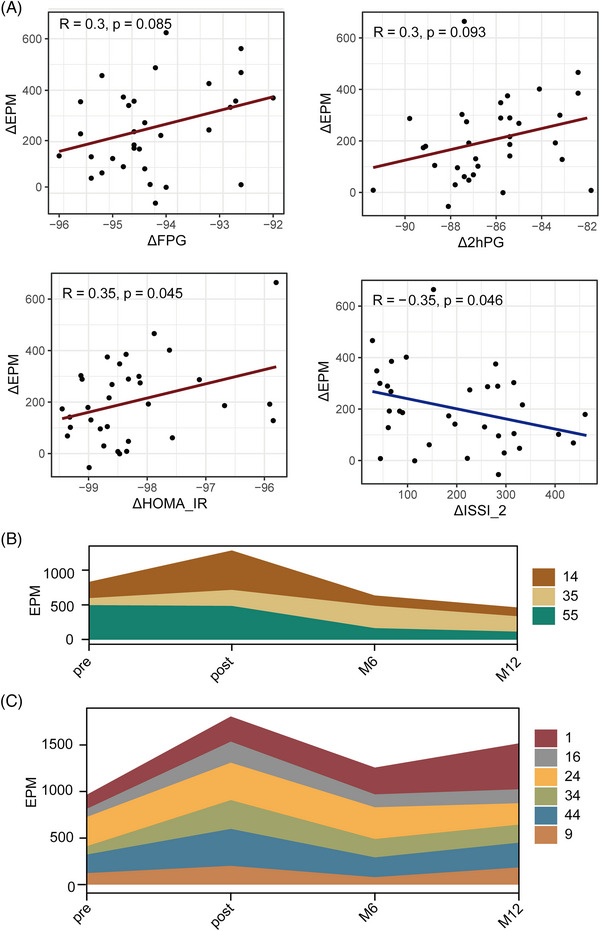
EPM, extrachromosomal circular DNA (eccDNA) count per million mappable reads, correlated with glycemic fluctuation during short‐term intensive insulin therapy (SIIT), and with diabetic status at 1‐year. (A) Dot plots between ΔEPM and ΔFPG, Δ2hPG, ΔHOMA‐IR and ΔISSI‐2, with lines as trend lines and correlation constants and their significance in the upper left corner. (the calculation of ΔValue, see the method). (B, C) Dynamics of patients' eccDNA profiles according to the status of type 2 diabetes mellitus (T2DM) for the patients under lifestyle intervention, pre‐SIIT, post‐SIIT, M6 (6 months after SIIT), and M12 (1 year after SIIT). EPM changes in remission patients (B), and non‐remission patients (C).

We extensively explored the plasma eccDNA profiles at three distinct time points: prior to SIIT, post‐SIIT and 1‐year post‐SIIT. Our results revealed a notable correlation between the dynamics of eccDNA and glycemic status. To evaluate the consecutively changing substances in plasma during SIIT treatment, we quantified a series of clinical measurements related to diabetes (see methods). To elucidate the correlation between eccDNA and diabetes status, we scrutinized the correlation between delta EPM (eccDNA read per million reads) and clinical parameter variations of diabetes during the SIIT process (Figure S3). It is found that delta EPM was positively correlated with delta FPG (R = 0.30, *p* = 0.085), delta 2hPG (R = 0.30, *p* = 0.093), and delta HOMA‐IR (R = 0.35, *p* = 0.045) (Figure 5A). Notably, delta EPM was negatively correlated with delta ISSI‐2 (R = 0.35, *p* = 0.046) (Figure 5A), where ISSI‐2 is a key parameter used to assess β‐cell function.^7^

Next, we intend to explore the potential application of eccDNA profile for the prediction of long‐term outcomes of SIIT. After SIIT, nine patients remained off hypoglycemic medication for 1 year, with follow‐up visits at 6 and 12 months. Among them, three were in remission criteria at 12 months, six were in non‐remission. Interestingly, in comparison with remission T2DM patients, patients with reappeared hyperglycemia exhibited a higher EPM at post‐SIIT than at pre‐SIIT (Figure 5B,C). For remission cases, their EPM showed a relatively large decrease after 1 year and was generally lower than their baseline before treatment (Figure 5B). For non‐remission patients, the EPM declined slightly after 1 year and was generally higher than their baseline level (Figure 5C). Collectively, our findings strongly suggest that plasma eccDNA could serve as a promising marker for monitoring disease progression and therapeutic response in diabetes.

## 4 DISCUSSION

In this study, we present the first comprehensive characterization of the plasma eccDNA landscape in patients with T2DM treated with SIIT. Meanwhile, we highlighted the potential utility of eccDNAs to predict the treatment response in New‐DM patients undergoing SIIT.

By applying the Circle‐Seq method, millions of eccDNA were detected from our 35 pairs of pre‐SIIT and post‐SIIT samples. Intriguingly, we identified a higher abundance of eccDNAs, as measured by EPM (eccDNA per million mappable reads), in the low‐glycemia samples (post‐SIIT) in comparison to the high‐glycemia samples (pre‐SIIT). This discrepancy may stem from the metabolic alterations occurring during the intense insulin therapy over the course of 1 week. In addition, we found that the plasma of T2DM patients exhibited considerable eccDNA load heterogeneity and shared similar eccDNA characteristics in terms of size, GC content, and distribution across chromosomes, which is consistent with previous observations.^14^, ^15^

Type 2 Diabetes Mellitus (T2DM) is characterized by a complex interplay of genetic, epigenetic, and environmental factors that lead to insulin resistance and pancreatic beta‐cell dysfunction. This multifaceted nature of T2DM involves altered glucose homeostasis, lipid metabolism, inflammation and various signalling pathways.^30^ Given the complexity of T2DM's pathogenesis and the diverse responses to treatments like SIIT, the identification and validation of specific biomarkers become essential to provide insights into individual disease progression, optimize therapeutic strategies and enable early detection and personalized care. Biomarkers in T2DM are an evolving field, covering traditional, genetic, and epigenetic indicators. Established biomarkers such as HbA1c and fasting glucose are central to clinical diagnosis.^31^ Additionally, research has investigated cfDNA, including differentially methylated circulating DNA, as an indicator of beta‐cell apoptosis in T2DM.^9^ Specific methylated genes have also been explored as potential biomarkers in T2DM, shedding light on epigenetic modifications related to insulin resistance and metabolic dysfunction.^32^ Recently, eccDNAs have been recognized in other diseases for their potential diagnostic value, suggesting a future avenue of exploration in T2DM. While the exact role of eccDNAs in T2DM remains to be elucidated, their involvement in other metabolic and regulatory processes hints at the possibility of their utility as a novel biomarker.^24^

Previous research has reported that cfDNA, a composite of both linear and eccDNA, primarily originates from apoptotic cells across various tissues, which release these molecules into the bloodstream.^15^, ^33^ It is found that approximately 30% of eccDNAs were derived from eccGenes, and about 50% of eccDNAs in the plasma of lung adenocarcinoma patients were eccGenes.^15^ This observation underscores the presence of distinct patterns of plasma eccDNAs in the pathophysiological of diverse diseases. Of note, recent studies have indicated that eccDNAs predominantly originate from genetically dense chromosomal regions.^27^ In line with these insights, we conducted an analysis focused on the gene sequences of our eccDNAs. Our findings revealed a genetic bias in the formation of eccDNA, which was evident in the context of different FPG levels. Specifically, genes such as IGFBP6, POU4F2, LEFTY2, CARS1, LCN10, HSPA8, DNAJB9, SOX8, ADCYAP1 and TCHHL1 were observed to produce eccDNA exclusively in the pre‐SIIT group. Remarkably, the ADCYAP1 gene reduces the apoptosis of pancreatic β‐cells by blocking apoptosis.^34^ Conclusively, these insights into the genetic predisposition for eccDNA formation shed light on the potential mechanisms underlying the observed changes in eccDNA patterns in response to SIIT and the implications for diabetes‐related pathophysiology.

As reported in a recent study, an important origin of eccDNA is the product of cell apoptosis.^25^ According to the observation from Figure 5, 1 week of normalized glycaemia, the raised eccDNA (ΔEPM) is correlated with the changes of glycaemia or β‐cell function, especially the significant associations of plasma eccDNA (ΔEPM) with insulin resistance (ΔHOMA‐IR) and β‐cell dysfunction (ΔISSI‐2). ISSI‐2, an established indicator of the insulin secretion process, has previously been shown to be closely related to β‐cell function.^7^, ^35^ SIIT treatment induces notable alterations in glucose levels and other metabolites, leading to corresponding changes in eccDNA levels in plasma.

To investigate the long‐term plasma eccDNA after SIIT, all patients were followed up for 1 year. Some of them were treated with various hypoglycemic drugs, which might influence the eccDNA by diverse mechanisms. To address this, we specifically analyzed the patients who received only lifestyle intervention for 1 year. Among them, three patients in remission displayed lower EPMs at 1‐year visit, whereas six non‐remission patients had higher EPMs along with a relapse of hyperglycemia. Interestingly, we saw an increased EPM at 1‐week's short‐term of dramatic glycemic control and a lower EPM at 1‐year's long‐term of euglycemic maintenance. This trend indicates that the increased EPM after SIIT could be attributed to the delayed clearance of eccDNA, although this hypothesis should be further validated with a larger sample size. Taken together, these observations suggest that hyperglycemia (Diabetes) and the pronounced fluctuation in glucose levels may indeed influence eccDNA levels.

In summary, this study represents the pioneering effort to unveil the dynamic behaviour of eccDNA in T2DM patients across different phases: pre‐SIIT, post‐SIIT and 1 year after SIIT (Figure S4). Our findings underscore the strong correlation between eccDNA and changes in glycemia, as well as the efficacy of 1‐year glucose control.

However, it's important to acknowledge the limitations imposed by the relatively small sample size used in this study. As a consequence, definitive causal or predictive conclusions regarding the potential of eccDNA as a biomarker remain challenging to establish. To address this limitation, the next phase of our research aims to engage a broader cohort of volunteers with type 2 diabetes, sourced from multiple centres. Additional experiments are also planned to validate the relationships between eccDNA, β‐cell status, and endothelial pathophysiology, thereby offering further insights into the underlying mechanisms.

## CONFLICT INTEREST STATEMENT

The authors declare no conflict of interest.

## Supporting information

(A) Correlation of the number of start breakpoints and the number of end breakpoints in each gene (Person correlation test). The start and end breakpoints of each eccDNA refer to the upstream and downstream boundary of the circularized genomic region.
(B) Accumulation curve of plasma eccDNA frequency with eccDNA length.
(C) Comparison of the percentage of eccDNA with indicated length ranges (≥ 500 bp; 500 bp ‐ 2 kb; 2 kb ‐ 10 kb; > 10 kb) in pre‐SIIT and post‐SIIT (Wilcox test).

(A) IGV depicted for eccDNAs span exons. The top of each IGV map was marked with the sample name, the gene ID in which the exon is located, and the coordinates of the eccDNA. The black bar on the bottom line represents for exon region, the thick line means the exon region and the thin line means the intronic region.
(B) PCR validation of eccDNAs span exons.
(C) Junction sites of eccDNAs span exons were obtained after the sequencing of PCR products.

The diagonal line shows the distribution of the variable itself; the lower triangle (lower left of the diagonal line) shows the scatter plot of the two variables; the upper three small shapes (upper right of the diagonal line), the number indicates two attributes The correlation value of, and the model indicates the degree of significance (more stars indicate more significance).

(A) Before the application of short intensive insulin treatment (pre‐SIIT), elevated glucose levels induce a mild inflammatory response, consequently affecting endothelial cells and various other cell types, leading to exacerbated intracellular gene fragment loss, and increased eccDNA loads. (B) During SIIT, there is a sustained reduction in glycemia. This undermines the existing glucose homeostasis. It influences the body's metabolism, inflammation, and modifications in various signalling pathways. These changes result in the worsening of gene fragment loss across multiple cells. There is a surge in eccDNA formation. Concurrently, eccDNA gradually enters the systemic circulation through multiple pathways and is detected. (C) Post‐SIIT, the glucose levels gradually stabilize and the internal metabolism, inflammation and other alterations also progressively recover. The intracellular genomic status also tends towards stabilization, and the production of eccDNA reverts to normal levels. However, the eccDNA generated during the treatment process is still slowly released into the blood circulation by the cells, resulting in a higher concentration of eccDNA in the patient's plasma post‐SIIT compared to pre‐SIIT. (D) After 1 year of lifestyle interventions, the pancreatic function of the patient achieves relative normalization, the concentration of glucose stabilizes and the quantity of eccDNA in the plasma also reverts to normal levels.

Supporting Information

TABLE S1 Primers for outward PCR and Sanger sequencing of eccDNA with sequences of *C20orf203* validated in Figure 4C.
**TABLE S2** Clinical data of T2DM patients not taking antidaibetic medication for 12 months.
**TABLE S3** Sequencing criteria of all samples in this study.
**TABLE S4** EccDNA number, eccDNA per million mappable reads, GC median, and coverage per sample.
**TABLE S5** Average normalized ratio of plasma eccDNA reads to repeat sequences between pre‐SIIT and post‐SIIT groups.
**TABLE S6** The 522 differential eccGenes between pre‐ and post‐SIIT groups in Figure 3A.
**TABLE S7** The 31 eccGenes which uniquely presented in pre‐ and post‐SIIT groups.
**TABLE S8** List of 135 unique eccDNAs carry exons of genes (Fisher's exact test).

## Data Availability

The raw sequence data reported in this paper have been deposited in the China National Center for Bioinformation, Chinese Academy of Sciences (GSA: HRA004303) and the CNGB Sequence Archive (CNSA) of China National GeneBank DataBase (CNP0003396) that are publicly accessible at https://ngdc.cncb.ac.cn/gsa and https://db.cngb.org/.
