# Supplementary figures and images for: Plasma extrachromosomal circular DNA is a pathophysiological hallmark of short‐term intensive insulin therapy for type 2 diabetes
Source: Clin Transl Med. 2023 Oct 20;13(10):e1437. doi: 10.1002/ctm2.1437 (PMC10587738; doi:10.1002/ctm2.1437)

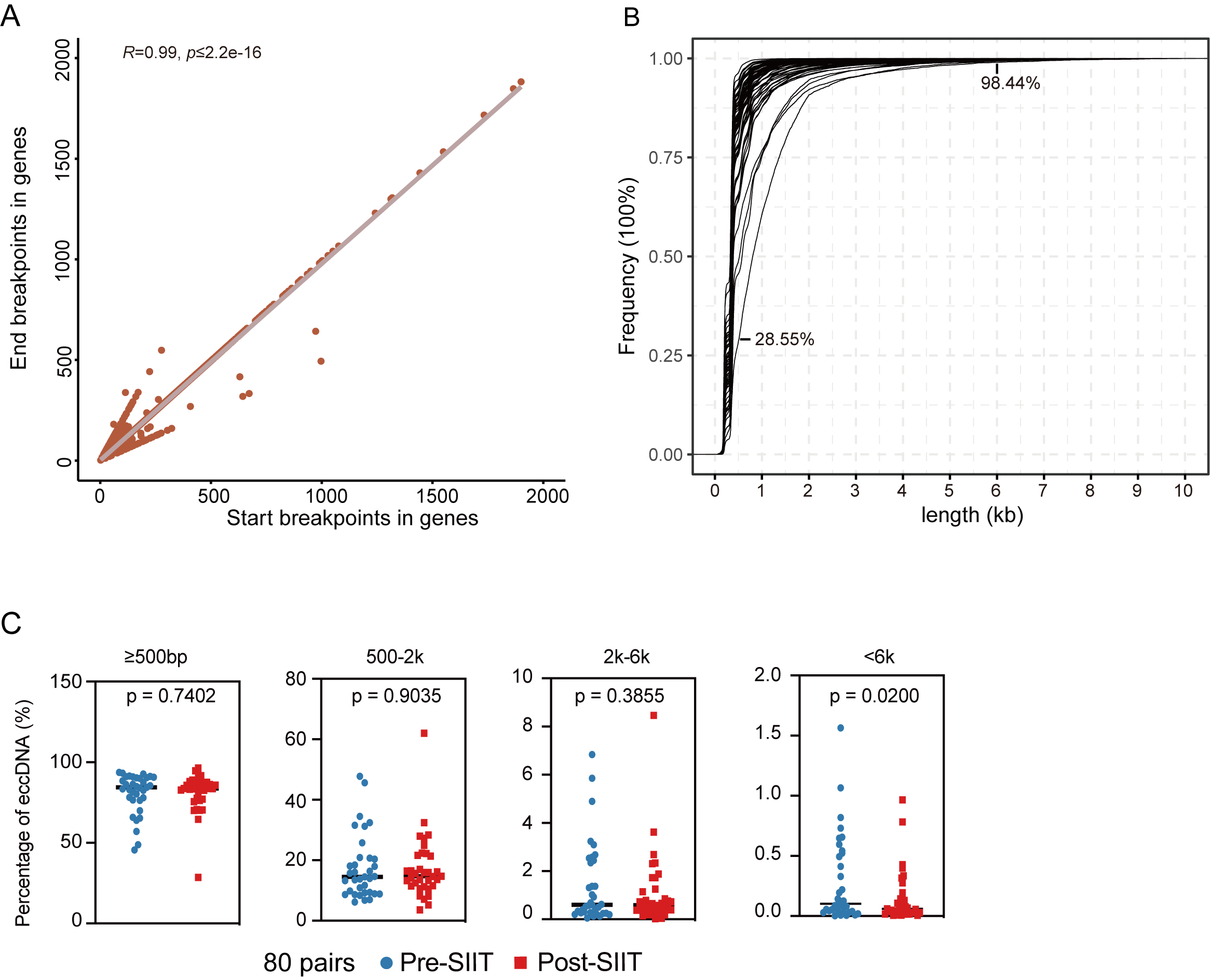

Supplement: Supplementary file 1 — (A) Correlation of the number of start breakpoints and the number of end breakpoints in each gene (Person correlation test). The start and end breakpoints of each eccDNA refer to the upstream and downstream boundary of the circularized genomic region. (B) Accumulation curve of plasma eccDNA frequency with eccDNA length. (C) Comparison of the percentage of eccDNA with indicated length ranges (≥ 500 bp; 500 bp ‐ 2 kb; 2 kb ‐ 10 kb; > 10 kb) in pre‐SIIT and post‐SIIT (Wilcox test). [file CTM2-13-e1437-s003.png]

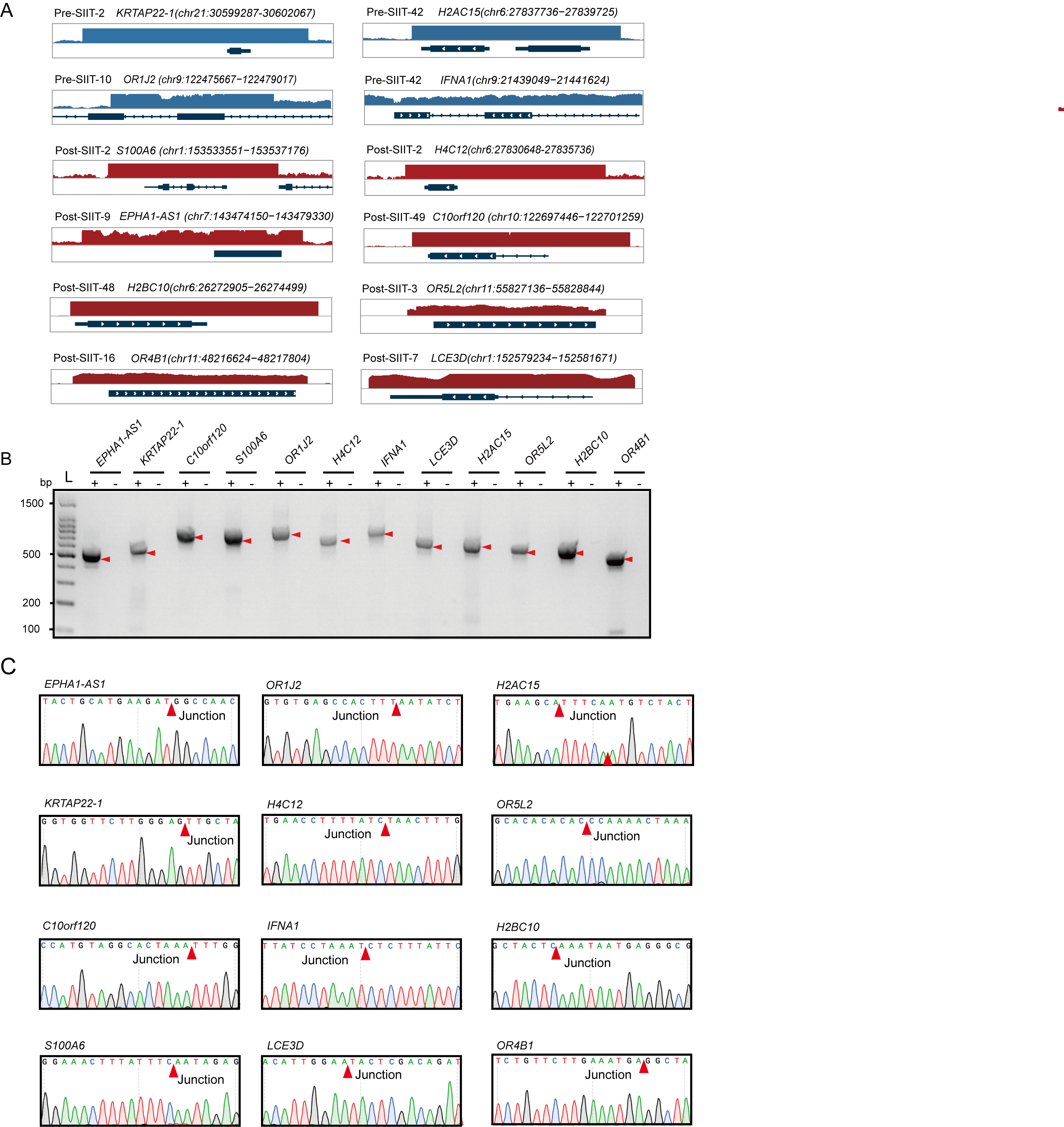

Supplement: Supplementary file 2 — (A) IGV depicted for eccDNAs span exons. The top of each IGV map was marked with the sample name, the gene ID in which the exon is located, and the coordinates of the eccDNA. The black bar on the bottom line represents for exon region, the thick line means the exon region and the thin line means the intronic region. (B) PCR validation of eccDNAs span exons. (C) Junction sites of eccDNAs span exons were obtained after the sequencing of PCR products. [file CTM2-13-e1437-s004.png]

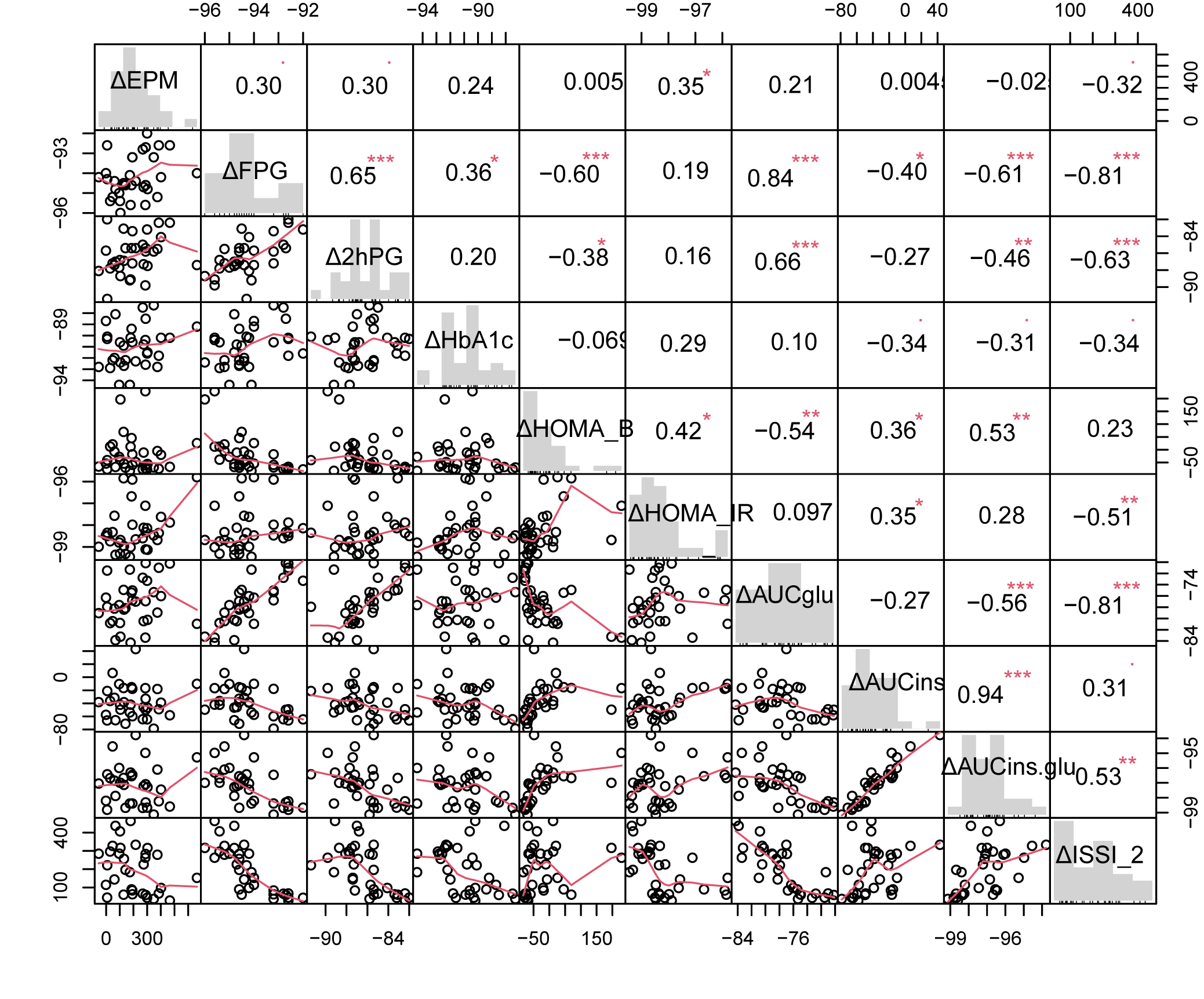

Supplement: Supplementary file 3 — The diagonal line shows the distribution of the variable itself; the lower triangle (lower left of the diagonal line) shows the scatter plot of the two variables; the upper three small shapes (upper right of the diagonal line), the number indicates two attributes The correlation value of, and the model indicates the degree of significance (more stars indicate more significance). [file CTM2-13-e1437-s006.png]

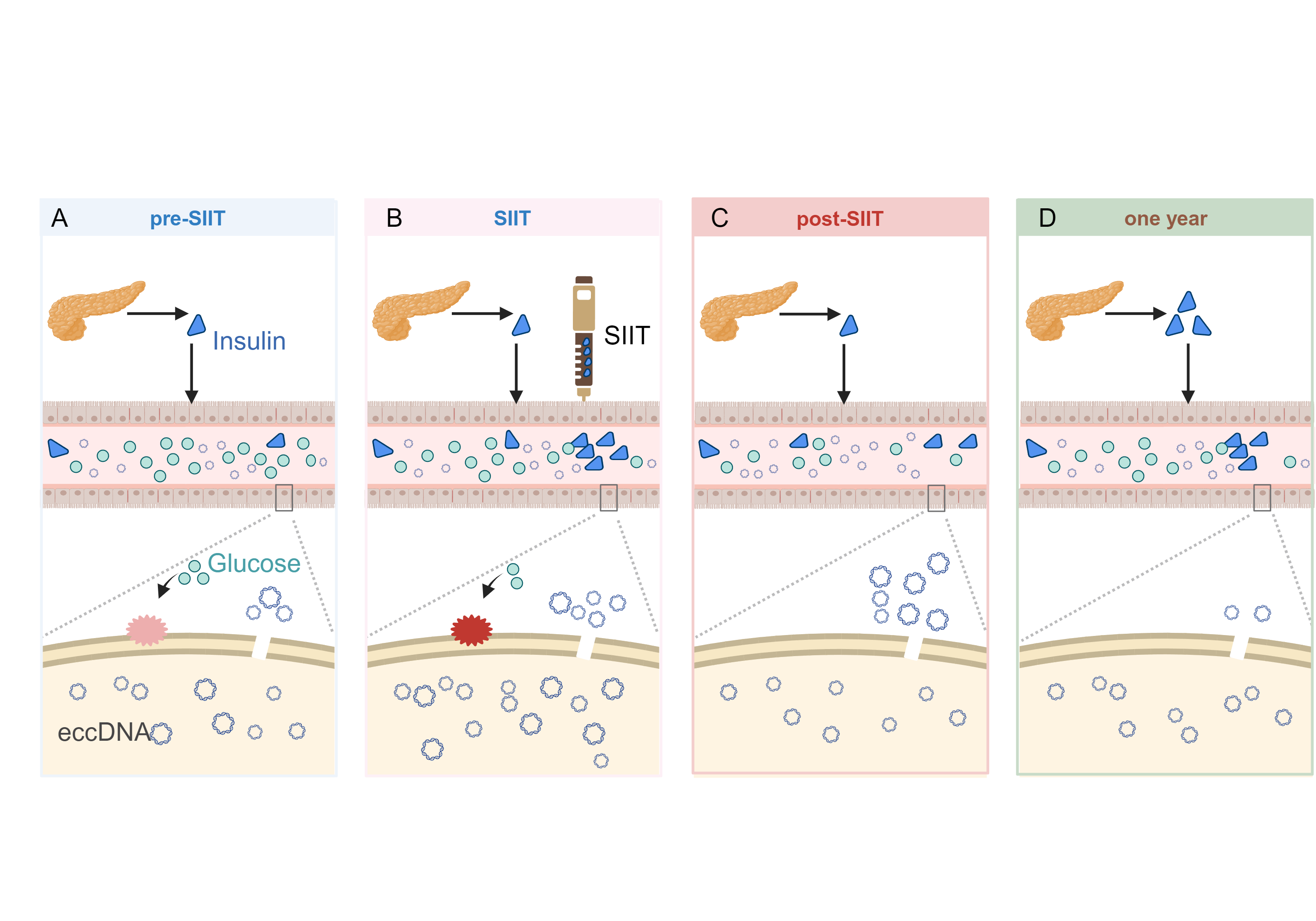

Supplement: Supplementary file 4 — (A) Before the application of short intensive insulin treatment (pre‐SIIT), elevated glucose levels induce a mild inflammatory response, consequently affecting endothelial cells and various other cell types, leading to exacerbated intracellular gene fragment loss, and increased eccDNA loads. (B) During SIIT, there is a sustained reduction in glycemia. This undermines the existing glucose homeostasis. It influences the body's metabolism, inflammation, and modifications in various signalling pathways. These changes result in the worsening of gene fragment loss across multiple cells. There is a surge in eccDNA formation. Concurrently, eccDNA gradually enters the systemic circulation through multiple pathways and is detected. (C) Post‐SIIT, the glucose levels gradually stabilize and the internal metabolism, inflammation and other alterations also progressively recover. The intracellular genomic status also tends towards stabilization, and the production of eccDNA reverts to normal levels. However, the eccDNA generated during the treatment process is still slowly released into the blood circulation by the cells, resulting in a higher concentration of eccDNA in the patient's plasma post‐SIIT compared to pre‐SIIT. (D) After 1 year of lifestyle interventions, the pancreatic function of the patient achieves relative normalization, the concentration of glucose stabilizes and the quantity of eccDNA in the plasma also reverts to normal levels. [file CTM2-13-e1437-s005.png]
