# Supplementary material for: Plasma extrachromosomal circular DNA is a pathophysiological hallmark of short‐term intensive insulin therapy for type 2 diabetes
Source: Clin Transl Med. 2023 Oct 20;13(10):e1437. doi: 10.1002/ctm2.1437 (PMC10587738; doi:10.1002/ctm2.1437)
Supplement: Supplementary file 5 — Supporting Information [file CTM2-13-e1437-s002.docx]

Table S2. Clinical data of T2DM patients not antidiabetic medication for 12 months.

|  | Remission at 1 year （n=3） | | | non-remission at 1year（n=6） | | |
| --- | --- | --- | --- | --- | --- | --- |
|  | Pre-SIIT | Post-SIIT | 1 year | Pre-SIIT | Post-SIIT | 1year |
| Age | 66.33±2.08 | | | 52.83±9.02 | | |
| ﻿Sex (male/female) | 1/2 | | | 4/2 | | |
| FPG（mmol/L） | 10.47±3.96 | 6.73±1.15 | 6.3±0.8 | 10.85±1.65 | 5.7±1.31^a^ | 8.45±1.68^b^ |
| HbA1c（%） | 9.77±1.82 | 8.43±1.27 | 6.6±0.26 | 9.08±0.79 | 7.82±1.22 | 6.75±0.63^b^ |
| HOMA-β | 21.28±12.59 | 34.85±10.18^a^ | 46.82±2.81 | 16.51±7.34 | 100.54±111.13 | 57.2±24.73^b^ |
| HOMA-IR | 2.98±1.95 | 1.6±0.54 | 1.87±0.7 | 2.81±1.4 | 1.68±1.29 | 5.26±2.82 |
| ﻿AUCglu | 29.63±5.83 | 26.78±4.52 | 20.48±0.26 | 31.33±5.78 | 23.33±4.45^a^ | 23.93±4.62^b^ |
| ﻿AUCins | 29.6±14.58 | 51.54±10.01^a^ | 52.33±8.82^b^ | 38.56±13.8 | 68.69±39.82^a^ | 65.8±23.41^b^ |
| AUC_ins/glu_ | 1.1±0.76 | 2.01±0.76^a^ | 2.56±0.46^b^ | 1.28±0.55 | 3.03±1.75 | 2.83±1.05^b^ |
| ISSI-2 | 115.15±89.66 | 217.46±121.97 | 285.75±94.38 | 113.18±58.04 | 308.99±132.99 ^a^ | 155.47±54.78 ^b^ |

Note: Continuous parametric data are presented as means ± SD. Continuous nonparametric variables are presented as medians (interquartile ranges). And categorical data were presented as proportion. Abbreviations: SIIT: short-term intensive insulin therapy; FPG: fasting plasma glucose; HbA1c: glycated hemoglobin; HOMA-β: homeostasis model assessment of insulin secretion; HOMA-IR: homeostasis model assessment of insulin resistance; AUCglu: the area under the glucose curve during a standard food-load test; AUCins: the area under the insulin curve during a standard food-load test; AUC_ins/glu_: AUC_ins_/AUC_glu_ ratio.

ISSI-2 : AUCins/glu multiplied by the Matsuda index.

a: p<0.05 comparison of Pre-SIIT and Post-SIIT; b: p<0.05 comparison of Pre-SIIT and 1-year.
